# Limited permissibility of ENL-R and Mv-1-Lu mink cell lines to SARS-CoV-2

**DOI:** 10.3389/fmicb.2022.1003824

**Published:** 2022-10-12

**Authors:** Marion Le Bideau, Gabriel Augusto Pires de Souza, Celine Boschi, Jean-Pierre Baudoin, Gwilherm Penant, Priscilla Jardot, Florence Fenollar, Philippe Colson, Matthias Lenk, Bernard La Scola

**Affiliations:** ^1^Microbes, Evolution, Phylogénie et Infection (MEPHI), Aix-Marseille Université, Institut de Recherche pour le Développement (IRD), Assistance Publique - Hôpitaux de Marseille (AP-HM), Marseille, France; ^2^Institut Hospitalo-Universitaire (IHU) Méditerranée Infection, Marseille, France; ^3^Vecteurs – Infections Tropicales et Méditerranéennes (VITROME), Aix Marseille Univ, Institut Hospitalo-Universitaire (IHU), AP-HM, Marseille, France; ^4^Collection of Cell Lines in Veterinary Medicine (CCLV), Department of Experimental Animal Facilities and Biorisk Management, Friedrich-Loeffler-Institut, Greifswald-Insel Riems, Germany

**Keywords:** mink, ENL-R, Mv-1-Lu, SARS-CoV-2, COVID-19, omicron

## Abstract

The SARS-CoV-2 pandemic started in the end of 2019 in Wuhan, China, which highlighted the scenario of frequent cross-species transmission events. From the outbreak possibly initiated by viral spill-over into humans from an animal reservoir, now we face the human host moving globally while interacting with domesticated and peridomestic animals. The emergence of a new virus into the ecosystem leads to selecting forces and species-specific adaptations. The adaptation of SARS-CoV-2 to other animals represents a risk to controlling the dissemination of this coronavirus and the emergence of new variants. Since 2020, several mink farms in Europe and the United States have had SARS-CoV-2 outbreaks with human–mink and mink–human transmission, where the mink-selected variants possibly hold evolutionary concerning advantages. Here we investigated the permissibility of mink lung-derived cells using two cell lines, Mv-1-Lu and ENL-R, against several lineages of SARS-CoV-2, including some classified as variants of concern. The viral release rate and the infectious titers indicate that these cells support infections by different SARS-CoV-2 lineages. The viral production occurs in the first few days after infection with the low viral release by these mink cells, which is often absent for the omicron variant for lung cells. The electron microscopy reveals that during the viral replication cycle, the endomembrane system of the mink-host cell undergoes typical changes while the viral particles are produced, especially in the first days of infection. Therefore, even if limited, mink lung cells may represent a selecting source for SARS-CoV-2 variants, impacting their transmissibility and pathogenicity and making it difficult to control this new coronavirus.

## Introduction

In December 2019, the Severe Acute Respiratory Syndrome Coronavirus-2 (SARS-CoV-2) emerged in Wuhan city, Hubei Province, China (Lai et al., 2020; Liu et al., 2020; Lu H. et al., 2020). This new coronavirus is responsible for the Coronavirus Disease-2019 (COVID-19), a viral pneumonia with varying degrees of severity (Huang et al., 2020; Liu et al., 2020; Zhu et al., 2020), which has rapidly spread worldwide (Huang et al., 2020), hitting a pandemic state as declared by the World Health Organization (WHO) in March 2020 (World Health Organization, 2020). In early September 2022, there have been 603,711,760 confirmed cases of COVID-19, including 6,484,136 deaths, globally reported to the WHO (WHO, 2021).

The family *Coronaviridae* includes several viruses that can infect humans and other vertebrates, and it is divided into four subfamilies, namely, alpha, beta, delta, and gamma-coronavirus (Chen et al., 2020). The animal reservoir of alpha- and beta-coronaviruses is mainly bats, while delta and gamma are of mainly birds and pigs (Murgolo et al., 2021). The beta-coronaviruses include the highly pathogenic SARS-CoV-1, Middle East respiratory syndrome-coronavirus (MERS-CoV), and the recently discovered SARS-CoV-2 (Zhu et al., 2020; Murgolo et al., 2021).

The SARS-CoV-2 pandemic highlighted a scenario of frequent cross-species transmission events. From an outbreak possibly originally initiated from viral spill-over into humans (Lam et al., 2020; Zhou et al., 2020), likely from an animal reservoir, we are now dealing with a human host that can move globally and interact with domesticated species and peridomestic animals (Bashor et al., 2021). Humans, therefore, carry the risk of reinserting viruses into nonhuman species, forcing them to adapt to new hosts, which typically results in species-specific adaptations (Sauter and Kirchhoff, 2019).

With a wide range of animals potentially susceptible to SARS-CoV-2, the role of these species as reservoirs for continued viral transmission remains unclear (Eckstrand et al., 2021). In animal studies, SARS-CoV-2 had a poor replication in dogs, pigs, chickens, and ducks but was efficiently replicated in cats. In cats, there are reports of both cat-to-cat (Shi et al., 2020; Hoffmann et al., 2021) and human-to-cat transmission of SARS-CoV-2 (Segalés et al., 2020).

Due to the need to predict potential hosts for SARS-CoV-2, the permissibility of cells from different species to this virus has been extensively tested (Wurtz et al., 2021). The SARS-CoV-2 looks to be able to infect/replicate in human, nonhuman primate, rabbit, pig, and cat cell lines (Chu et al., 2020). This type of analysis is essential as these evolutionary changes resulting from the adaptation to new hosts can determine the pathogenicity and transmissibility of the virus in novel host species (Andersen et al., 2020).

Since 2020, several mink farms in Europe and the United States have had SARS-CoV-2-confirmed outbreaks with human–mink and mink–human transmission (Eckstrand et al., 2021; Hammer et al., 2021; Shriner et al., 2021). In May 2020, in the Netherlands, four mink farms were affected by COVID-19. Besides animals presenting respiratory symptoms, SARS-CoV-2 was detected in pharyngeal and lung samples from mink (Oreshkova et al., 2020). These mink-selected variants showed evolutionary advantages, such as preliminary results that pointed to weak reactions to human neutralizing anti-SARS-CoV-2 antibodies (Frutos and Devaux, 2020; Fenollar et al., 2021; Devaux et al., 2021b). Therefore, the adaptation of SARS-CoV-2 to other animals represents a risk to controlling the dissemination of the SARS-CoV-2 and the emergence of new variants.

Here we investigated the permissibility of mink lung cells using two cell lines, the Mv-1-Lu and the ENL-R, both derived from mink lung tissues, against several lineages of SARS-CoV-2, including some of the ones classified as variants of concern by the WHO. The Mv-1-Lu cell has already been described as a cell that expresses the Angiotensin Converting Enzyme-2 (ACE2) receptor, responsible for interacting with the SARS-CoV-2 spike (S) protein during the first step of the viral entry process, and as permissive cells for SARS-CoV (Azkur, 2020; Hoffmann et al., 2020; Stout et al., 2021). Mv-1-Lu is, therefore, a potential SARS-CoV-2 permissive cell line. In contrast, the ENL-R cell line will undergo inoculation of coronavirus for the first time.

## Materials and methods

### Cell line cultures

Mv-1-Lu (CCLV-RIE 0048, Friedrich-Loeffler-Institut) and ENL-R (CCLV-RIE 0240, Friedrich-LoefflerInstitut) cells are mink lung cells. These two cell lines were cultured and maintained in M10 medium [minimum essential medium (MEM)]. Gibco, Thermo Fisher Scientific with 10% fetal bovine serum (FBS; Thermo Fisher Scientific), 2 mM L-glutamine (L-Gln: Thermo Fisher Scientific), and NaHCO_3_ (Sigma-Aldrich), adjusted to contain 850 mg/L. For the ENL-R cells, M10 medium was added 1% MEM nonessential amino acids solution (NEAA, Thermo Fisher Scientific). The medium was replaced by a fresh medium twice a week.

Calu-3 cells (ATCC® HTB-55TM) are human lung epithelial cells, and Vero E6 cells (ATCC CRL-1586TM) are African green monkey kidney epithelial cells that exhibit contact inhibition. Both cell lines were cultured and maintained in an M10 medium. Once a week, the medium was changed. Mv-1-Lu, ENL-R, Calu-3, and Vero E6 cells were maintained in 175 cm^2^ culture flasks in the absence of antibiotics at 37°C with 5% CO_2_. After reaching confluence, the cells were subsequently subcultured by trypsinization.

### Preparation of standardized viral suspensions on Vero E6 cells

Vero E6 cells were cultured in 24-well flat-bottom plates (Ref. 11874235, Thermo Fisher Scientific) at a density of 5 × 10^5^ cells/mL in M10 cell culture medium (1 ml/well) and incubated for 24 h at 37°C under 5% CO_2_. Upon reaching confluence, Vero E6 monolayers were infected with 200 μl of each SARS-CoV-2 strain diluted at 1:10. Finally, 24 h later, the viral suspensions were harvested and filtered through a 0.2-μm pore filter. The quantification of the viral suspensions was carried out by real-time RT-qPCR specifically targeting the N gene (Smyrlaki et al., 2020). The viral suspensions were diluted to obtain a standardized viral load calibrated at 20 Ct. The entire virus culture work was performed in a biological safety cabinet in a biosafety level 3 laboratory.

### Performing the culture test on Mv-1-Lu and ENL-R cells

One day before the tests, the two Mv-1-Lu and ENL-R cell lines were inoculated in 24-well plates at 2 × 10^5^ cells/mL and 5 × 10^5^ cells/ml, respectively, at a volume of 1 ml per well, in their specific growth medium and incubated for 24 h at 37°C under 5% CO_2_. At this stage, the cells were subconfluent and were infected with 200 μL of the standardized viral inoculum for each strain as described previously. Negative controls were carried out by adding 200 μL of specific growth medium to the two cell lines without any viral suspension. These plates were centrifuged for 1 h at 3,452 × g, and all wells were rinsed three times in their respective culture medium. A total of 100 μL was collected from each well at H0. Then, the plates were incubated at 37°C under 5% CO_2_ and a volume of 100 μL was collected from each well 1 and 7 days postinfection (d.p.i), to perform RT-qPCRs.

### Detection of viral growth

RT-qPCRs were performed on the 100 μL of the viral suspensions collected. First, RNA was extracted using the QIAamp 96 Virus QIAcube HT kit (Qiagen), according to the manufacturer’s procedure. To detect SARS-CoV-2 RNA, RT-qPCRs targeted the N gene, using the previously described primers (Lu X. et al., 2020): forward: GACCCCAAAATCAGCGAAAT, reverse: TCTGGTTACTGCCAGTTGAATCTG and probes FAM-ACCCCGCATTACGTTTGGTGGACC-QSY. The RT-qPCRs were carried out using the Superscript III Platinum One-step Quantitative RT-qPCR systems with ROX kit (Invitrogen) following the manufacturer’s recommendations with adding the RNaseOUT, with a final concentration of 400 nM of primers, of 200 nM of the probe, in a final volume of 25 μL with 2 μL of RNA. The RT-qPCR program is that described by the manufacturer. The RT-qPCRs were carried out on a LightCycler 480 I (Roche Diagnostics). The ΔCt (Ct 0 d.p.i–Ct 1 d.p.i) and (Ct 0 d.p.i–Ct 7 d.p.i) were calculated. The heatmaps were built by Morpheus^2^ after inserting a math matrix based on the ΔΔCt between the strains, where ΔΔCt = [(Average of ΔCt_Strain Y_) − (Average of ΔCt_Strain X_)].

### Virus production and titration in Mv-1-Lu, ENL-R, Calu-3, and Vero E6 cells

The titer of infecting viral particles on the Mv-1-Lu, ENL-R, Calu-3, and Vero E6 cell lines was quantified by determining the median infectious dose in tissue culture (TCID50; Ramakrishnan, 2016) and was calculated according to the Spearman–Kärber method after 0, 1, 2, 3, and 7 d.p.i. All the TCID50 assays were performed on Vero E6 cells by using supernatants of infected cell cultures, using four replicates per dilution. Briefly, ENL-R and Calc-3 at 2 × 10^5^ cells/ml and Mv-1-Lu and Vero E6 at 5 × 10^5^ cells/mL were seeded 1 day before the infection in 6-well plates in 5 ml of cell culture medium and infected with 800 μl of viral suspensions standardized to 20 Ct, as previously described, by using the following virus strains: IHU-MI 3, IHU-MI 2129, IHU-MI 3396, IHU-MI 5253, IHU-MI 5234, and IHU-MI 5227. Then, the plates were centrifuged for 1 h at 3,452 × g, rinsed three times, and incubated at 37°C with 5% CO_2_.

At each timepoint, 600 μl of cell culture supernatants was collected from each well and replaced with their respective fresh cell culture media. 200 μL of the supernatant was used for the RT-qPCRs. The remaining 400 μL was filtered through a 0.2 μm syringe filter and immediately diluted up to 10–8. Each dilution of the viral supernatants was inoculated on monolayers of Vero E6 cells, cultured the day before at a density of 5 × 10^5^ cells/ml per well in 96-well plates. Four replicates for each dilution of the virus supernatant were performed. The plates were incubated for 7 days to determine the TCID50/mL by the characteristic cytopathic effect (CPE) on Vero E6 cells. This procedure was performed on two independent experiments.

### Electronic microscopy

Similarly, 96-well plates with single break strip wells were made with Mv-1-Lu at 5 × 10^5^ cells/mL and ENL-R at 2 × 10^5^ cells/mL with 200 μL per well. They were then infected with 50 μL of viral suspensions calibrated at 20 Ct, centrifuged for 1 h at 3452 × g, and then rinsed three times. The infected wells were fixed on days 0, 1, 2, and 7 d.p.i with the addition of 20 μL of 25% glutaraldehyde. The wells thus fixed were prepared with a microwave-assisted resin coating directly in the wells, and ultra-thin sections were cut straight through the monolayers of the infected cells and then observed with SU5000 SEM (Hitachi High-Technologies, HHT, Japan; Le Bideau et al., 2021). This electron microscopic observation was performed on SARS-CoV-2 strains of groups B.1 and B.1.160. In addition, scanning electron microscopy of ultra-thin sections of Mv-1-Lu and ENL-R cell monolayer was performed according to the novel approach described by Le Bideau et al. (2021).

## Results

### Analysis of viral release rate on Mv-1-Lu and ENL-R cells

**Figure 1:**
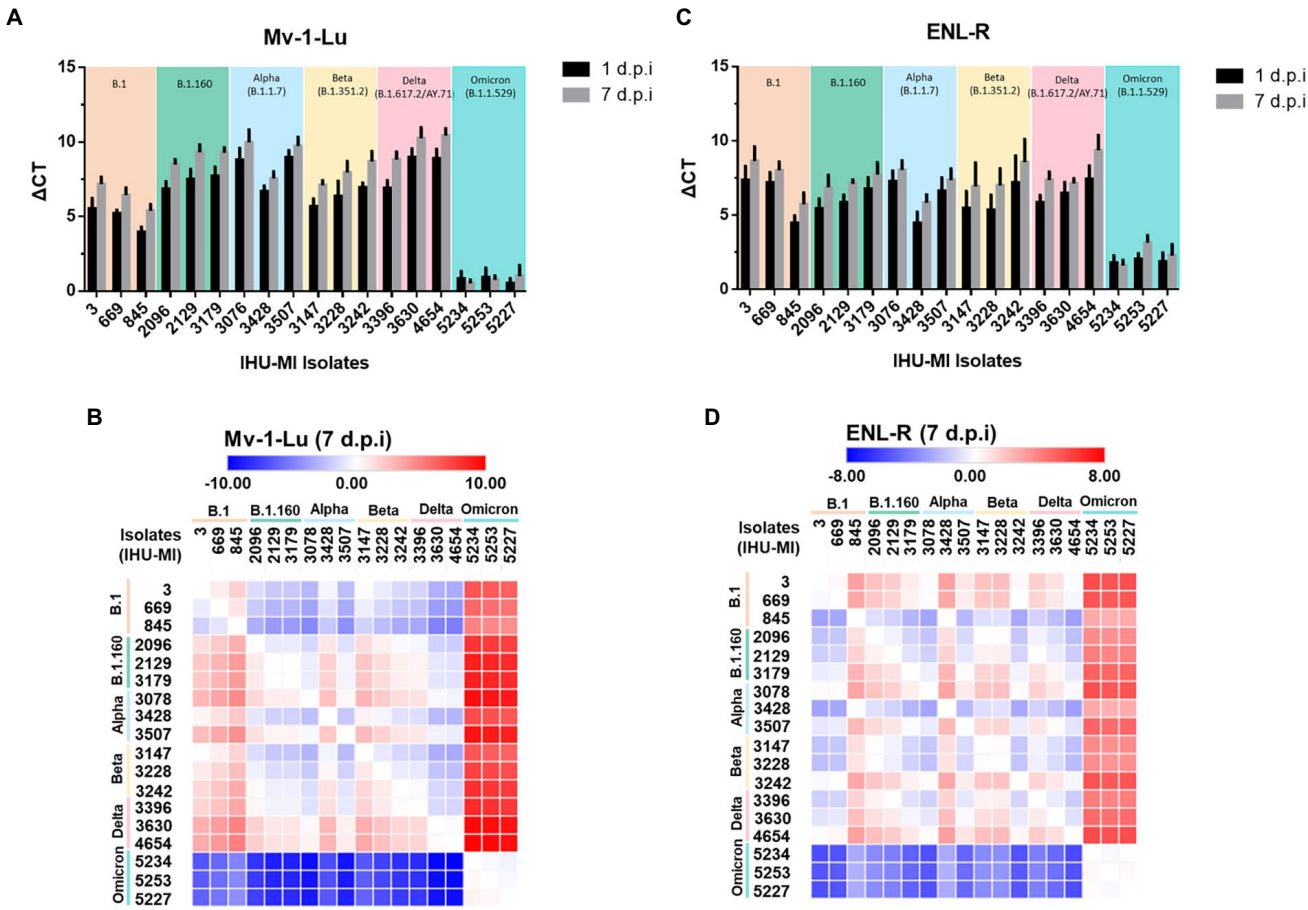
SARS-CoV-2 viral replication in mink cells. Replication rate of 18 isolates of SARS-CoV2 belonging to six different lineages in two cell lines derived from mink lungs. The viruses have been grouped based on the PANGO classification. The replication rate is expressed in ΔCt (cycle threshold) between time 0 and 1 days postinfection (black) and between time 0 and day 7 post-infection (gray); **(A)** Replication rate on the Mv-1-Lu cell line; **(B)** Heatmap comparing SARS-CoV-2 variant replication on Mv-1-Lu mink cells 7 days post infection; **(C)** Replication rate on the ENL-R cell line; **(D)** Heatmap for SARS-CoV-2 variant replication on ENL-R mink cells 7 days post infection.

The analysis of the viral release rate of the 18 strains of SARS-CoV-2 was made based on the viral genome relative quantification by RT-qPCR of the mink cellular supernatant collected at 1 and 7 d.p.i. First, the strains were grouped according to their respective lineage. It was observed that none of the strains showed a high difference on the viral release between the first and the seventh day postinfection whether for the Mv-1-Lu cells (Figure 1A) or ENL-R (Figure 1C), revealing that the viral release is minimal or even interrupted after at least the first 24 h of infection. However, when the strains were grouped according to their lineage, differences in viral release patterns on mink cells between SARS-CoV-2 lineages and strains from the same lineage were evidenced (Figure 1).

In Mv-1-Lu cells, the strains from the B.1 lineage (Table 1) had a lower viral release independently of the day than those from the other lineages, except when compared to Omicron. No Omicron strain seems to replicate in Mv-1-Lu cells. Curiously, IHU-MI 845 (B.1) and 3428 (Alpha) strains presented a lower viral release than the two other isolates of their respective lineage, demonstrating a difference in the viral growth of isolates from the same genotype (Figures 1A,B).

**Table 1:** List of isolated SARS-CoV-2 and their respective genotypes and classifications.

Isolates (IHU-MI)	Lineage (PANGO)	Lineage (Nextclade)	WHO Label	CDC classification	Country first identified (community)
3	B.1	20A	–	–	China
669		20C			
845		20A			
2096	B.1.160	20A	–	–	Europe and United Kingdom (multiple countries)
2129					
3179					
3076	B.1.1.7	20I	Alpha	Variant of concern	United Kingdom
3428					
3507					
3147	B.1.351	20H	Beta	Variant of concern	South Africa
3228					
3242					
3396	B.1.617.2	21 J	Delta	Variant of concern	India
3630	AY.71				
4654	AY.4				
5234	BA.1	21 K	Omicron	Variant of concern	South Africa
5,253					
5227					

In the ENL-R, the viral release mean of all lineages was similar for 1 d.p.i (B.1_ΔCt_: 6.34 ± 1.46; B.1.160 _ΔCt_: 6.01 ± 0.79; alpha _ΔCt_: 6.12 ± 1.37; beta _ΔCt_: 5.99 ± 1.44; delta _ΔCt_: 6.59 ± 0.90), most were close to average replication, excluding omicron (ΔCt mean 6.12 ± 1.25). As observed in Mv-1-Lu cells, the strains from omicron do not seem to multiply in the ENL-R mink cell line, and the isolates IHU-MI 845 and 3,428 presented a lower viral release (Figures 1C,D). Also, in ENL-R cells, the isolates IHU-MI 3242 and 4654 presented higher viral release than other strains in their lineage (beta and delta, respectively).

### Analysis of the viral production in Mv-1-Lu, ENL-R, Calu-3, and Vero E6 cells lines

**Figure 2:**
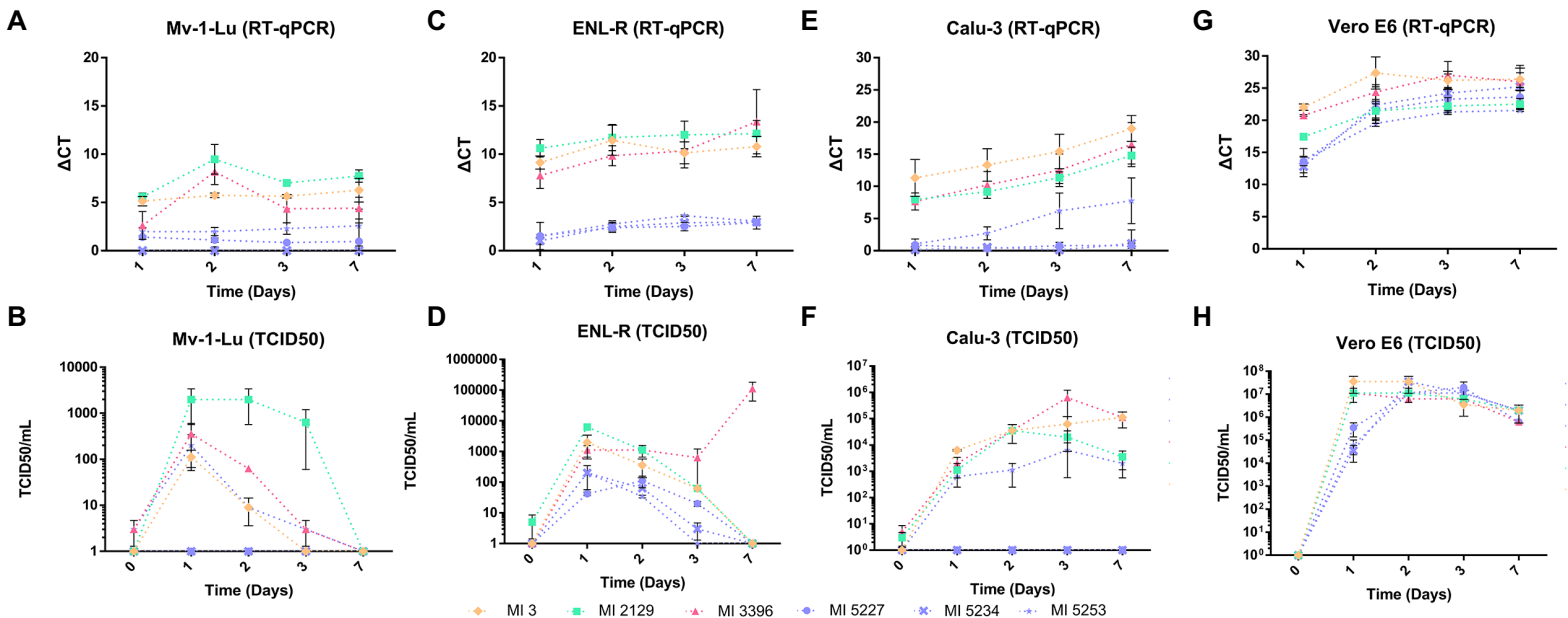
SARS-CoV-2 release and its viral titer over a week of infection in different cells. The viral release was defined by the relative quantification of the viral genome by RT-qPCR and the median tissue culture infectious dose (TCID50) of cell supernatants recovered from each cell line starting from 1 to 7 days postinfection (d.p.i). The ΔCt was calculated from the values obtained in the assay using one replicate from two different experiments. The TCID50/ml values were obtained from fresh supernatant inoculum into Vero E6 cells, using four replicates per dilution. **(A)** Genomic viral release quantification (ΔCt) from Mv-1-Lu cells supernatant; **(B)** Infective particle quantification (TCID50/ml) on Mv-1-Lu cell supernatant; **(C)** Genomic viral release quantification (ΔCt) from ENL-R cell supernatant; **(D)** Infective particle quantification (TCID50/ml) in ENL-R cells supernatant; **(E)** Genomic viral release quantification (ΔCt) from Calu-3 cell supernatant; **(F)** Infective particle quantification (TCID50/ml) in Calu-3 cell supernatant; **(G)** Genomic viral release quantification (ΔCt) from Vero E6 cell supernatant; **(H)** Infective particle quantification (TCID50/ml) in Vero E6 cell supernatant.

The infectious viral titer of six strains was monitored over 7 days by TCID50 and compared with the viral release observed by RT-qPCR from the culture supernatant on the following four different cell lines: two mink derived cells (Mv-1-Lu and ENL-R), one human derived cell (Calu-3), and one monkey derived cell (Vero E6; Figure 2; Table 2). The six strains selected belong to four distinct genotypes, namely, IHU-MI 3 (B.1), IHU-MI 2129 (B.1.160), IHU-MI 3396 (delta), and IHU-MI 5253/5234/5227 (omicron).

**Table 2:** Viral production of SARS-CoV-2 of different strains in mink lung cells over 7 days.

			Lineage					
			B.1	B.1.160	Delta	Omicron		
Cell Type	d.p.i	Strain	MI 3	MI 2129	MI 3396	MI 5227	MI 5234	MI 5253
Mv-1-Lu	0	TCD50/ml	0.00 ± 0.00	0.00 ± 0.00	3.00 ± 1.71	0.00 ± 0.00	0.00 ± 0.00	3.00 ± 1.71
		ΔCT	-	-	-	-	-	-
	1	TCD50/ml	(1.12 ± 0.46) × 10^2^	(1.99 ± 1.42) × 10^3^	(3.56 ± 2.45) × 10^2^	0.00 ± 0.00	0.00 ± 0.00	2.00 ± 1.43 × 10^2^
		ΔCT	5.14 ± 0.49	5.60 ± 0.38	2.61 ± 1.44	1.41 ± 0.04	0.29 ± 0.06	1.97 ± 0.41
	2	TCD50/ml	9.00 ± 5.4	(1.99 ± 1.42) × 10^3^	63.0 ± 0.00	0.00 ± 0.00	0.00 ± 0.00	9.00 ± 5.40
		ΔCT	5.74 ± 0.24	9.48 ± 1.54	8.18 ± 1.32	1.10 ± 0.22	−0.18 ± 0.59	1.98 ± 0.42
	3	TCD50/ml	0.00 ± 0.00	(6.32 ± 5.72) × 10^2^	3.00 ± 1.71	0.00 ± 0.00	0.00 ± 0.00	3.00 ± 1.71
		ΔCT	5.64 ± 0.18	7.02 ± 0.19	4.35 ± 1.48	0.84 ± 0.40	0.55 ± 0.52	2.3 ± 0.60
	7	TCD50/ml	0.00 ± 0.00	0.00 ± 0.01	0.00 ± 0.02	0.00 ± 0.03	0.00 ± 0.04	0.00 ± 0.05
		ΔCT	6.28 ± 1.235	7.74 ± 0.64	4.41 ± 1.12	0.96 ± 1.915	0.23 ± 0.74	2.58 ± 1.72
ENL-R	0	TCD50/ml	0.00 ± 0.00	5.00 ± 3.58	0.00 ± 0.00	0.00 ± 0.00	0.00 ± 0.00	0.00 ± 0.00
		ΔCT	-	-	-	-	-	-
	1	TCD50/ml	(1.99 ± 1.42) × 10^2^	(6.29 ± 0.00) × 10^3^	(1.12 ± 0.46) × 10^3^	9.00 ± 5.40	(2.00 ± 1.43) × 10^2^	(2.00 ± 1.43) × 10^3^
		ΔCT	9.14 ± 0.69	10.62 ± 0.91	7.79 ± 1.33	1.52 ± 0.14	1.02 ± 0.37	1.53 ± 1.40
	2	TCD50/ml	3.56 ± 2.16	(1.12 ± 0.46) × 10^3^	(1.12 ± 0.46) × 10^3^	(1.12 ± 0.46) × 10^2^	63.2 ± 0.00	9.00 ± 3.20
		ΔCT	11.45 ± 1.55	11.73 ± 1.36	9.84 ± 1.03	2.39 ± 0.48	2.49 ± 0.44	2.75 ± 0.36
	3	TCD50/ml	63.0 ± 0.00	63.0 ± 0.01	(6.32 ± 5.72) × 10^2^	5.00 ± 3.60	(3.00 ± 1.71)	0.00 ± 0.00
		ΔCT	10.14 ± 1.135	12 ± 1.425	10.34 ± 1.76	2.5 ± 0.44	2.89 ± 0.23	3.63 ± 0.07
	7	TCD50/ml	0.00 ± 0.00	0.00 ± 0.00	(1.12 ± 0.68) × 10^5^	0.00 ± 0.00	0.00 ± 0.00	0.00 ± 0.00
		ΔCT	10.79 ± 1.04	12.12 ± 1.36	13.37 ± 3.32	2.91 ± 0.67	2.98 ± 0.04	3.06 ± 0.13
Calu-3	0	TCD50/ml	0.00 ± 0.00	3.00 ± 1.71	0.00 ± 0.00	0.00 ± 0.00	0.00 ± 0.00	0.00 ± 0.00
		ΔCT	-	-	-	-	-	-
	1	TCD50/ml	(6.29 ± 0.41) × 10^3^	(1.12 ± 0.87) × 10^3^	(1.99 ± 1.42) × 10^3^	0.00 ± 0.00	0.00 ± 0.00	(6.29 ± 0.00) × 102
		ΔCT	11.32 ± 2.86	7.96 ± 0.08	7.66 ± 1.32	0.88 ± 0.21	0.13 ± 0.64	1.06 ± 0.76
	2	TCD50/ml	(3.54 ± 2.40) × 10^4^	(3.54 ± 2.40) × 10^4^	(3.54 ± 2.40) × 10^4^	0.00 ± 0.00	0.00 ± 0.00	(1.12 ± 0.87) × 103
		ΔCT	13.34 ± 2.52	9.17 ± 0.01	10.22 ± 2.09	0.31 ± 0.23	0.54 ± 0.24	2.71 ± 1.02
	3	TCD50/ml	(6.29 ± 5.72) × 10^4^	(1.99 ± 1.42) × 10^4^	(6.29 ± 5.72) × 10^5^	0.00 ± 0.00	0.00 ± 0.00	(6.29 ± 5.72) × 103
		ΔCT	15.42 ± 2.68	11.36 ± 0.91	12.51 ± 2.53	0.79 ± 0.12	0.10 ± 1.22	6.2 ± 2.76
	7	TCD50/ml	(1.12 ± 0.68) × 10^5^	(3.54 ± 2.40) × 10^3^	(1.12 ± 0.68) × 10^5^	0.00 ± 0.00	0.00 ± 0.00	(1990 ± 1423.5)
		ΔCT	18.99 ± 2.01	14.79 ± 1.32	16.51 ± 3.44	0.78 ± 0.41	1.12 ± 2.12	7.77 ± 3.54
Vero E6	0	TCD50/ml	0.00 ± 0.00	0.00 ± 0.00	0.00 ± 0.00	0.00 ± 0.00	0.00 ± 0.00	0.00 ± 0.00
		ΔCT	-	-	-	-	-	-
	1	TCD50/ml	(3.54 ± 2.43) × 10^7^	(1.12 ± 0.68) × 10^7^	(1.12 ± 0.68) × 10^7^	(3.54 ± 2.15) × 10^5^	(3.54 ± 2.44) × 10^4^	(6.29 ± 3.94) × 10^4^
		ΔCT	22.05 ± 0.48	17.44 ± 0.32	20.76 ± 0.12	13.6 ± 0.66	12.8 ± 1.58	13.73 ± 1.89
	2	TCD50/ml	(3.54 ± 2.43) × 10^7^	(1.12 ± 0.68) × 10^7^	(6.29 ± 0.00) × 10^6^	(1.12 ± 0.68) × 10^7^	(3.54 ± 2.44) × 107	(1.12 ± 0.68) × 107
		ΔCT	27.37 ± 2.50	21.46 ± 1.10	24.39 ± 1.16	21.56 ± 1.42	22.35 ± 2.85	19.52 ± 0.45
	3	TCD50/ml	(3.54 ± 2.43) × 10^6^	(6.29 ± 0.00) × 10^6^	(6.29 ± 0.00) × 10^6^	(1.99 ± 1.42) × 10^7^	(1.12 ± 0.68) × 10^7^	(1.12 ± 0.68) × 10^7^
		ΔCT	26.22 ± 1.4	22.2 ± 1.3	27.05 ± 2.1	23.3 ± 1.81	24.22 ± 2.98	21.3 ± 0.33
	7	TCD50/ml	(1.99 ± 1.42) × 10^6^	(1.99 ± 1.42) × 10^6^	(6.29 ± 0.00) × 10^5^	(6.29 ± 0.00) × 10^5^	(1.99 ± 1.42) × 10^6^	(1.99 ± 1.42) × 10^6^
		ΔCT	26.39 ± 1.73	22.52 ± 0.90	25.99 ± 1.4	23.62 ± 1.50	25.2 ± 3.34	21.54 ± 0.19

In Mv-1-Lu cellular type, the strains IHU-MI 2129 (B.1.160) and IHU-MI 3396 (delta) shared similar viral release rates, detected from the first day but with a peak at the second d.p.i and with a reduction in RNA detected from the third d.p.i (Figure 2A; Table 2). The viral release of the IHU-MI 3 strain (B.1) seemed to remain practically the same from the first day. The three omicron strains look to have a minimal release, even lower than the IHU-MI 3 (Figure 2A). No release was observed for IHU-MI 5234 (omicron).

As for the number of SARS-CoV-2 infectious particles produced by the Mv-1-Lu cells, only B.1.160 seemed to produce above 10^3^ TCID50/ml, remaining until after the third day, reaching zero at the seventh d.p.i (Figure 2B). The other isolates had no infectious particles detected from 3 d.p.i. In addition, two of the three omicron isolates (IHU-MI 5234 and 5227) did not present a production of infectious particles during these 7 days (Figure 2B).

For the second mink cellular type, the ENL-R (Figure 2C; Table 2), the strains IHU-MI 3 (B.1) and IHU-MI 2129 (B.1.160) lineages follow the same release profile detected in the first d.p.i (ΔCt 9.14 and 10.62, respectively) which remains practically stable until 7 d.p.i (Figure 2C). However, when analyzing the number of infectious particles, it seems to decrease over time for the strains from the B.1 and B.1.160 until no infectious particles were detected in 7 d.p.i when these strains were produced in ENL-R cells (Figure 2D). For the isolate IHU-MI 3396 (delta), the peak of viral release occurred on the seventh d.p.i (Figure 2C), when the highest concentration of infectious particles was also observed (1.35 × 10^4^; Figure 2D; Table 2). Viral production for the three strains of omicron was practically negligible, not reaching values higher than 2.00 × 10^2^ TCID50/ml (Figures 2C,D; Table 2).

The SARS-CoV-2 production of these six strains was also evaluated on Calu-3, a human cell line. Differently from what was observed, a gradual release of the virus was detected by RT-qPCR over the 7 days of infection for four of the six strains, except for the identical two omicron isolates (IHU-MI 5234 and 5227) that did not seem to be produced by Calu-3 cells, as observed for Mv-1-Lu (Figure 2E). The release rate followed by RT-qPCR of the B.1 strain (IHUMI-3) was higher than that of the others, as the B.1.160 (IHU-MI 2129) and delta (IHU-MI 3396) isolates had similar rates, while these both were higher than the single omicron (IHU-MI 5253) with replication detected in Calu-3.

As for viral production, as previously observed in the RT-qPCR, from the omicron strain isolates, only the IHU-MI 5253 isolate established viable viral progeny, with low production, reaching titers not greater than 7.0 × 10^3^ in the third d.p.i (Figure 2F; Table 2). All of the other strains reached 4.26 × 10^4^ TCID50/ml in the second d.p.i, with a decline in the isolate B.1.160 after 3 d.p.i, and 7 d.p.i for B.1 (Figure 2F; Table 2).

In the primate Vero E6, high replication rates and viral titers were detected for all strains evaluated between 1 and 2 d.p.i (Figures 2G,H). On the second day, the recovered titers were higher than 5.00 × 10^6^ for all isolates, and even after 7 days, they remained higher than 6.00 × 10^5^ (Figure 2H; Table 2). On this cell line, no difference was observed between the groups and between the strains of the same group.

### Morphological examination under an electron microscope was carried out on the six strains of SARS-CoV-2 of groups B.1 and B.1.1.160 of the panel

**Figure 3:**
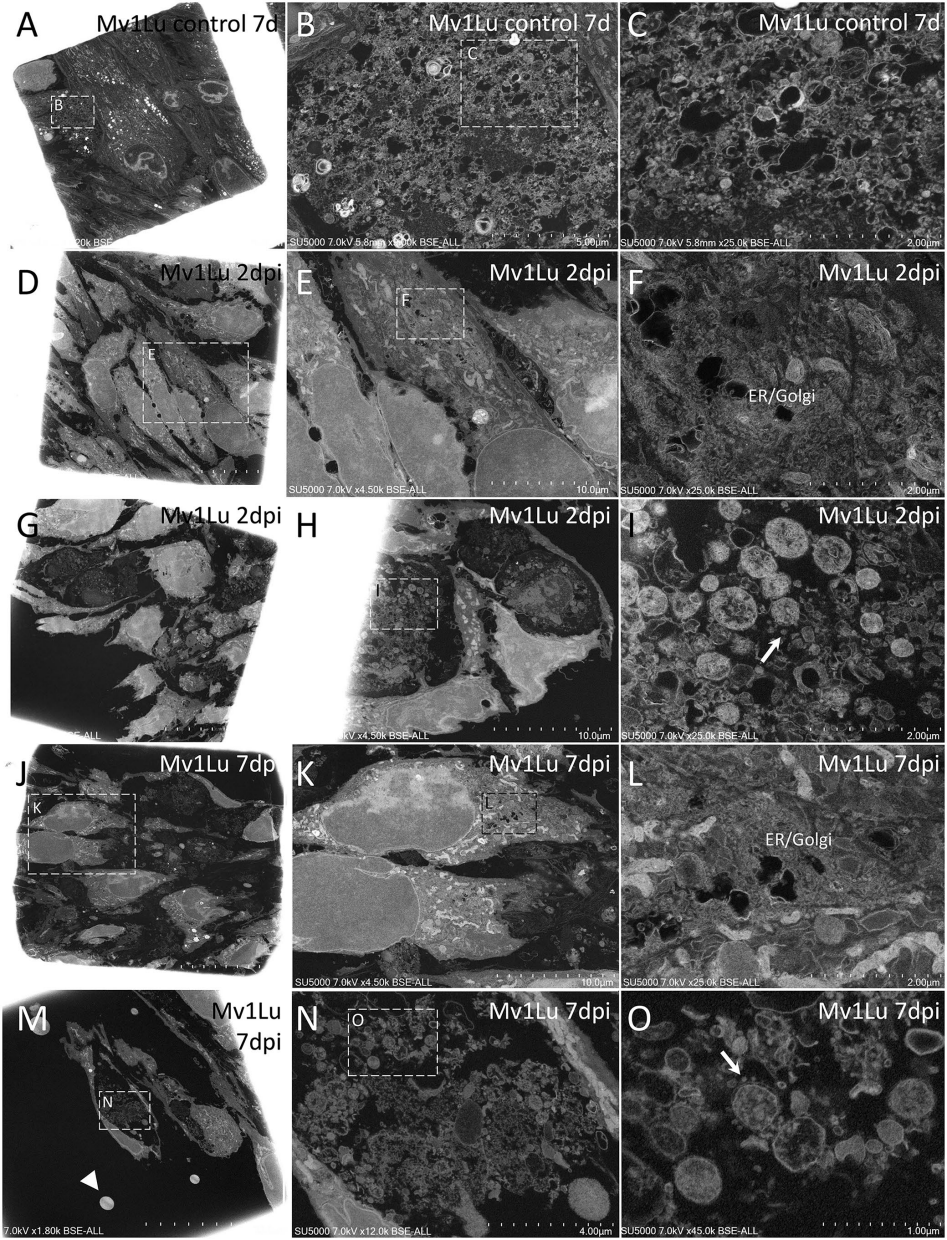
Scanning electron microscopy of SARS-CoV-2-infected Mv-1-Lu cell monolayer ultra-thin sections. **(A)** Low-magnification view of control, 7 days non-infected Mv-1-Lu cells monolayer. **(B)** Zoom-in boxed region in **(A)** with material between non-infected cells. **(C)** Zoom-in boxed region in **(B)**, with dense or empty objects, with diameters generally below 60 nm and above 140 nm. **(D)** Low-magnification view of Mv-1-Lu cell monolayer mostly intact at 2 d.p.i with SARS-CoV-2. **(E)** Zoom-in infected Mv-1-Lu cell boxed in **(D)**. **(F)** Zoom-in boxed region in **(E)** with an extended endoplasmic reticulum/Golgi apparatus. **(G)** Low-magnification view of Mv-1-Lu cell monolayer at 2 d.p.i with SARS-CoV-2 with lytic cells. **(H)** Lytic debris of 2 d.p.i infected Mv-1-Lu cells located among intact cells inside the monolayer. **(I)** Zoom-in boxed region in **(H)** with SARS-CoV-2 virions (arrow) located among extracellular cell debris. **(J)** Low-magnification view of Mv-1-Lu cell monolayer at 7 d.p.i with SARS-CoV-2 with lytic cells. **(K)** Zoom-in boxed region in **(J)** with two intact cells. **(L)** Zoom-in boxed region in **(K)** with an extended endoplasmic reticulum/Golgi apparatus. **(M)** Low-magnification view of Mv-1-Lu cell monolayer at 7 d.p.i with lytic debris and hyperdense nuclei (arrowhead). **(N)** Lytic debris from boxed region in **(M)**. **(O)** Zoom-in boxed region in **(N)** with SARS-CoV-2-like particles (arrow) among lytic debris.

**Figure 4:**
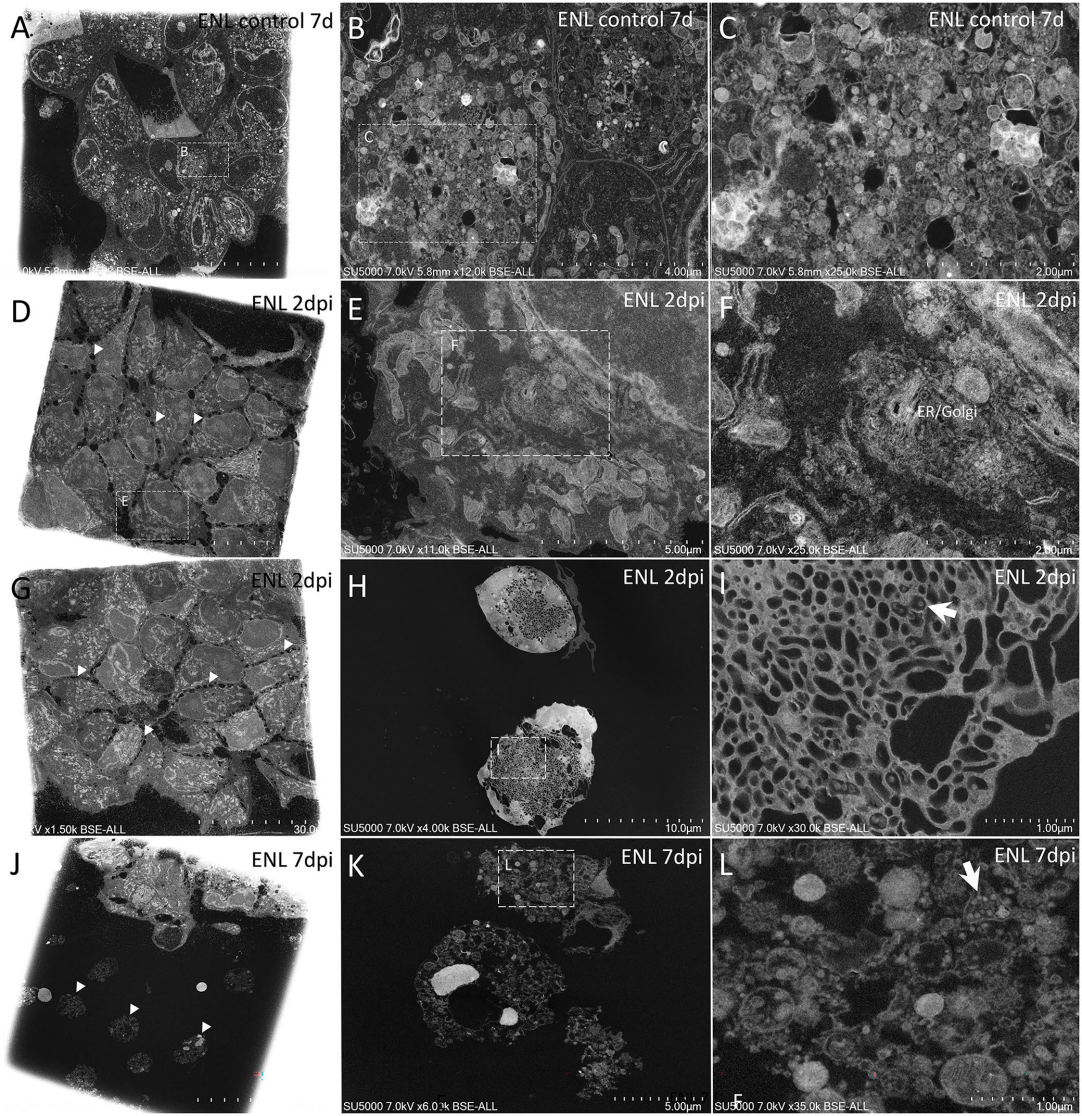
Scanning electron microscopy of SARS-CoV-2-infected ENL-R cell monolayer ultra-thin sections. **(A)** Low-magnification view of control, 7 days noninfected ENL-R cell monolayer; Cells are tightly attached to each other. **(B)** Zoom-in boxed region in **(A)** depicting large pockets of material inside control cells. **(C)** Material from boxed region in **(B)** with electron-dense or empty objects, with diameters generally below 60 nm and above 140 nm. **(D)** Low-magnification view of ENL-R cell monolayer at 2 d.p.i with SARS-CoV-2. Cells do not adhere to each other by clear membrane apposition but rather by sparse protrusions (arrowheads). **(E)** Zoom-in boxed cell in **(D)**. **(F)** Zoom-in boxed region in **(E)** with an extended endoplasmic reticulum/Golgi apparatus. **(G)** Low-magnification view of ENL-R cell monolayer at 2 d.p.i with SARS-CoV-2. Cells do not show clear confluency (arrowheads). **(H)** Foamy ENL-R cells at 2 d.p.i with SARS-CoV-2, excluded from the monolayer and presenting an extended tubulo-vesicular network. **(I)** SARS-CoV-2-like particles (arrow) from boxed regions in **(H)**. **(J)** Lyzed ENL-R cells at 7 d.p.i with SARS-CoV-2, with hyperdense nuclei (arrowheads). **(K)** Lyzed ENL-R cells at 7 d.p.i with SARS-CoV-2. **(L)** Zoom-in boxed region in **(K)** with cellular debris and SARS-CoV-2-like virions (arrow).

Non-infected Mv-1-Lu (Figures 3A–C) and ENL-R (Figures 4A–C) cells cultured for 7 days as controls. We observed dispersed material between cells and inside cells inside large vacuoles. This material was in these cases, mostly composed of membranes and small objects consisting in possible endoplasmic reticulum/Golgi apparatus-derived vesicles and glycogen granules with diameters in the 40–60 nm diameter range, as well as electron-dense lipid granules and cytoplasmic vesicles with diameters above 140 nm. We also noticed fibrillary material around elongated non-infected Mv-1-Lu cells, resembling mucus, as was the case for SARS-CoV-2 infected Mv-1-Lu cells. In the 7 d.p.i ENL-R cell monolayers, cells were closely attached to each other *via* membrane apposition with only a few free spaces between contacting cells.

At 2 d.p.i, Mv-1-Lu cells were, for most of them, intact, with an elongated morphology, and juxtaposed rather than contacting each other, with no clear membrane apposition (Figures 3D,E,G,H). Regarding the replication cycle in Mv-1-Lu cells, we observed an extended endoplasmic reticulum (ER)/Golgi apparatus (GA) network (Figures 3D–F). A few lyzed Mv-1-Lu cells were found among the intact cells, and possible SARS-CoV-2 particles of 90–100 nm diameter were found among the lytic debris (Figures 3G,H), as identified in SARS-CoV-2-infected Vero cells (Brahim et al., 2020). We observed loose contacts between cells in the monolayer for ENL-R cells *via* sparse protrusions (Figures 4D,G). We also observed for ENL-R cells a few proliferations of the ER/GA network (Figures 4D–F). SARS-CoV-2 –like particles were distributed among lytic ENL-R cell debris, and these materials were surrounded by intact ENL-R cells. In addition, a few ENL-R cells presented canaliculi containing SARS-CoV-2 –like particles (Figures 4G–I), giving the appearance of round, foamy cells. Thus, at 2 d.p.i, SARS-CoV-2 replication appeared rare in the two cell lines, with completely damaged lyzed cells for both and an also foamy cell for ENL-R cells.

At 7 d.p.i, we observed in Mv-1-Lu cells potential virus factories in intact cells with large ER/GA networks (Figures 3I–L), as well as lyzed Mv-1-Lu cells and SARS-CoV-2-like particles among debris between intact cells, forming large lytic pockets in the cell monolayer (Figures 3M–O). We also noticed dispersed single hyperdense nuclei (Figure 3M). For ENL-R cells, we observed a few SARS-CoV-2–like particles located among lytic material (Figures 4J–L) and hyper-dense nuclei (Figures 4J,K). We did not observe virus-producing ENL-R cells at 7 d.p.i.

## Discussion

The Mv-1-Lu cells were expected to be SARS-CoV-2 permissive cells (Figure 1A), first as previously understood permissive cells for the SARS-CoV in studies during the early 20th-century outbreak (Gillim-Ross et al., 2004). Mv-1-Lu cells are mink fetal lung-derived cells permissive to several viral species (e.g., vaccinia virus, herpes simplex virus, reovirus 3, e, and influenza) and are widely used by diagnostic laboratories to detect these viruses (Gillim-Ross et al., 2004). With the perspective that Mv-1-Lu cells could successfully replicate SARS-CoV, the hypothesis emerged that mink and other related species are potential animal models or natural reservoirs (Martina et al., 2003; Gillim-Ross et al., 2004; Kuba et al., 2005).

Evidence pointing to this hypothesis was also reported during the SARS-CoV-2 pandemic, since in 2020, numerous mink farms, both in Europe and the United States, had confirmed SARS-CoV-2 outbreaks with human–mink and mink–human transmission (Oreshkova et al., 2020; World Health Organization, 2020; Eckstrand et al., 2021; Shriner et al., 2021). Furthermore, these mink-selected variants showed evolutionary advantages, such as preliminary results that pointed to weak reactions to human neutralizing anti-SARS-CoV-2 antibodies (Frutos and Devaux, 2020; Fenollar et al., 2021; Frutos et al., 2021; Devaux et al., 2021a).

With the emergence of several variants of SARS-CoV-2, many of them being classified as variants of concern by the WHO, there is also a need to investigate the implications for viral fitness (ability to infect humans/animal cells) of these different variants. Therefore, if minks are considered as potential reservoirs of bidirectional SARS-CoV-2 infections, investigating the affinity of circulating variants with cells from this animal seems to be a plausible strategy.

In Mv-1-Lu cells, isolates from B.1 strains, although they could be produced by these cells, had a reduced expression compared to isolates from the strains B.1.160, alpha (B.1.1.7), beta (B.1.351.2), and delta (B.1.637.2/AY.7). The B.1 lineage represents isolates from the beginning of the pandemic, with viral isolates closest to the strain isolated in Wuhan in the initial outbreak in 2019, which later spread around the world in early 2020. Therefore, these isolates have few mutations: the isolates IHU-MI 669 and IHU-MI 845 have only one single mutation (D614G) in the spike (S) structural protein (de Souza et al., 2022).

The later strains have acquired evolutionary advantages that allow a more efficient replication in Mv-1-Lu cells, except for the variant of omicron isolates. In contrast to what was observed in Mv1-Lu cells, in ENL-R cells, no strain appeared to have a greater or lesser adaptation to these cells, except the omicron strains (Figures 1C,D). As previously observed in Mv-1-Lu cells, the omicron variant does not appear to replicate in these cells either. ENL-R cells are also derived from mink lung tissues, consisting of a non-homogeneous population with several cell types, including epithelioid cells. The heterogeneity of ENL-R cells is possibly why the production of SARS-CoV-2 is lower in ENL-R than in the other purely epithelial cells tested.

The omicron variant was first identified in South Africa and Botswana. However, it spreads rapidly globally, being classified as a variant of concern (VOC) by the WHO on November 26, 2021 (Burki, 2022; Fan et al., 2022). There are three hypotheses proposed for the development of the omicron variant, namely, (i) silent evolution in a population with little sequencing; (ii) long-term evolution in one or a few persons with chronic infection, or (iii) that this variant evolution occurred in another animal host, especially rodents (Kupferschmidt, 2021; Fan et al., 2022; Mallapaty, 2022). Significantly this last hypothesis reinforces the need to monitor other SARS-CoV-2 host animals and their potential for the diffusion of new variants.

Mounting evidence, mainly from animal studies, suggests that omicron does not multiply readily in lung tissue (Abdelnabi et al., 2022; Halfmann et al., 2022; McMahan et al., 2022), which explains the absence or the poor viral release of the strains in the mink Mv-1-Lu and ENL-R (Figures 1, 2A–D), but also in the human lung derived cell Calu-3 (Figures 2E,F). Other evidence also points out that the omicron variant may have switched of entering route to use endosomal fusion through cathepsins instead (Willett et al., 2022). The SARS-CoV-2 Wuhan 1 strain (B lineage) was used to estimate the relative usage of entry pathways in different cell lines. It demonstrated that each cell lineage has a relative percentage of entry preferential pathway mediated by host proteases to be used by the virus (Padmanabhan et al., 2020). Using the omicron variant (B.1.1.529), another study presented the increase of cathepsin B/L mediated entry compared to other strains (Padmanabhan and Dixit, 2022—Preprint). This change in the entry route may impact cells with high expression of TMPRSS2 and explain the lower affinity of this variant for lung cells.

The analysis of the viral release by RT-qPCR 1 and 7 d.p.i in Mv-1-Lu and ENL-R cells indicated no significant increase in viral production after the first day of infection (Figures 1A,C). However, as these cells have no cytopathic effect for SARS-CoV-2 infection (Supplementary Figure 2), viral production was monitored over a week by detection of the genome and viral particles in the supernatant recovered from the mink cells (Mv-1-Lu and ENL-R) and compared with the production of a human cell also derived from lung (Calu-3) and with monkey kidney cells (Vero E6), typically used in the isolation and production of SARS-CoV-2.

The isolates representing the B.1, B.1.160, and delta strains appeared to have peaks of release between 1 and 2 days of infection (Figures 2A,C). Still, the released particles remained viable for a short time because the low viral titers decreased day by day. Omicron’s minimal production in ENL-R cells followed this same pattern (Figures 2C,D). In Calu-3 cells, perceived as highly permissive to SARS-CoV-2 infection (Chu et al., 2020; Lee et al., 2020) the strains produced remained being released until the seventh day, and even if the viral titers dropped during the long incubation period (Figures 2D,F), they did not reach zero, as in mink cells.

Unlike lung cells, Vero E6 cells showed a high production of SARS-CoV-2 on the first day after infection that remained with high titers even on the seventh day (Figures 2G,H). Vero E6 cells are widely used in SARS-CoV-2 stocks due to the high recovered titers (Ogando et al., 2020), having a production typically higher than that obtained in Calu-3 cells (de Souza et al., 2022). Because all strains employed were isolated and produced in Vero E6 cells, it is plausible that these viruses are better adapted to this cell lineage. Furthermore, studies indicate that successive passages in Vero rapidly lead to attenuation of SARS-CoV-2 strains’ attenuation of a mink-associated SARS-CoV-2 variant (Cluster 5) as previously reported (Ogando et al., 2020; Lamers et al., 2021; Lassaunière et al., 2021). Therefore, attenuation by passages in Vero cells should be considered when evaluating strains in other cell lines.

As previously observed, the B.1 strain replicates less in mink cells than the strains of the lineages B.1.160 and delta (Figures 1, 2A–F). Especially the B.1.160 lineage has been suggested as a lineage originated from minks (Fournier et al., 2021). Although it reaches higher levels of particles released in the cell supernatant of Calu-3 cells, the number of infectious particles remains similar among the three strains (B.1, B.1.160, and delta; Figures 2E,F). This is yet another indication that the strain closest phylogenetically to the Wuhan strain, which started the SARS-CoV-2 outbreak in 2019, is more adapted to human lung cells than to the lungs of other hosts.

The new strains would also acquire greater adaptability to other hosts with the accumulation of mutations. Sustained infection and transmission of SARS-CoV-2 in a new animal host, as the minks, allows evolutionary changes to occur by selecting new variants with potential consequences for transmission, pathogenicity, and, as presented in this work, cell fitness (Lassaunière et al., 2021).

Considering this aspect between variants, we sought to characterize the replication of two phylogenetically close lineages (B.1 and B.1.160) but different replication rates in the two mink cells (Mv-1-Lu and ENL-R). For Mv-1-Lu cells, morphological changes were present in the infected cells, both in organelles such as the endoplasmic reticulum and Golgi apparatus (Figures 3A,B) and in the presence of vesicles in virus-producing cells (Figures 3C–F).

It is a common strategy for vertebrate positive-strand RNA viruses to remodel the endomembrane system of the host cell (Lee and Ahlquist, 2003; Den Boon and Ahlquist, 2010). Analyses of coronaviruses factories present a reticulovesicular network of modified endoplasmic reticulum (ER) integrating convoluted membranes, numerous double-membrane vesicles (DMVs), and vesicle packets that arise from the merging of DMVs (Knoops et al., 2008; Grangeon et al., 2012; Brahim et al., 2020; Barreto-Vieira et al., 2022). Due to the biogenesis of these virus factories, the host secretory pathway function is affected.

The morphological changes promoted by the viral infection often trigger the cytopathic effect (Albrecht et al., 1996), which, although it was not visible when observing the monolayer of cells in ordinary light microscopy, in electron microscopy, the infected cells were often lysed after infection, for both Mv-1-lu (Figures 3C–F) and ENL-R (Figure 4A) cells. The SARS-CoV-2 particles were identified based on previous electron microscopy morphological studies from our laboratory and other groups (Brahim et al., 2020; Colson et al., 2020; Laue et al., 2021; Le Bideau et al., 2021).

However, we did not observe large vacuoles containing SARS-CoV-2 virions like those observed in the Vero E6 cell (Brahim et al., 2020). On the contrary, it is observed that although CPE is not evident in the monolayer of ENL-R cells, in scanning electron microscopy analyses, the loss of cell–cell contact is suggested, similarly to what is observed in Caco-2 cells infected with SARS-CoV-2 (Osman et al., 2022). In Caco-2, a cell line also lacks CPE for SARS-CoV-2; this reduced contact in infected cells was attributed to E-Cadherin cleavage as a consequence of viral infection (Osman et al., 2022). The cleavage of E-Cadherin would impair the maintenance of cell contact junctions.

The results obtained in electron microscopy are congruent with those obtained by viral release analysis, both observed by RT-qPCR and TCID50. Viruses released by infected cells do not infect adjacent cells, which explains the full release observed between 1 and 2 d.p.i in mink cells (Figures 2A–D) and the absence of virus production on the seventh day. In ENL-R cells, no viral particles were detected in formation on the seventh day, which explains the no-changing release observed in the RT-qPCR of the cellular supernatant (Figure 2C).

Considering these aspects, both Mv-1-Lu and ENL-R cells can be classified as susceptible and permissive to SARS-CoV-2. They are susceptible to infection that triggers morphological changes in cellular organelles to allow the release of viable infectious particles, even though these are limited to the early periods of infection. Therefore, although it is difficult to establish patterns of viral replication according to SARS-CoV-2 strains due to the heterogeneity observed between isolates of the same genotype, it is frequent for several strains. There is a limited production of infective particles by Mv-1-Lu and ENL-R, both cell lines derived from mink lung, when infected with different strains of SARS-CoV-2. These cells can therefore be characterized as cells permissive to SARS-CoV-2, presenting alterations typically triggered by the replication of coronaviruses in the endomembrane system of the host cell. They suggest that SARS-CoV-2 could establish animal reservoirs in minks, which can select new variants, impacting their transmissibility and pathogenicity and making it difficult to control this new coronavirus.

## Data availability statement

The raw data supporting the conclusions of this article will be made available by the authors, without undue reservation.

## Author contributions

BS: conceptualization, validation, supervision, and project administration. BS and J-PB: methodology. GAPS, J-PB, and MB: formal analysis. GAPS, J-PB, CB, GP, PJ, and MB: investigation. GAPS, J-PB, and MB: data curation. GAPS and MB: writing—original draft preparation. FF, PC, ML, and BS: writing—review and editing. All authors contributed to the article and approved the submitted version.

## Funding

This work was supported by the French Government under the “Investments for the Future” program managed by the National Agency for Research (ANR), Méditerranée-Infection 10-IAHU-03 and was also supported by Région Provence-Alpes-Côte d’Azur and European funding ERDF PRIMMI (European Regional Development Fund - Plateformes de Recherche et d’Innovation Mutualisées Méditerranée Infection).

## Conflict of interest

The authors declare that the research was conducted in the absence of any commercial or financial relationships that could be construed as a potential conflict of interest.

## Publisher’s note

All claims expressed in this article are solely those of the authors and do not necessarily represent those of their affiliated organizations, or those of the publisher, the editors and the reviewers. Any product that may be evaluated in this article, or claim that may be made by its manufacturer, is not guaranteed or endorsed by the publisher.

## Supplementary material

The Supplementary material for this article can be found online at: https://www.frontiersin.org/articles/10.3389/fmicb.2022.1003824/full#supplementary-material

SUPPLEMENTARY FIGURE 1 Heatmap of replication rate among different SARS-CoV-2 isolates in mink lung cells 1-day post-infection. **(A)** Mv-1-Lu cells **(B)** ENL-R.

SUPPLEMENTARY FIGURE 2 Absence of cytopathic effect on mink lung cells after 7 days post-infection with SARS-CoV-2 (IHU-MI 3 Strain).

## Footnotes

^1^ http://software.broadinstitute.org/morpheus
