# Supplementary figures and images for: Limited permissibility of ENL-R and Mv-1-Lu mink cell lines to SARS-CoV-2
Source: Front Microbiol. 2022 Oct 12;13:1003824. doi: 10.3389/fmicb.2022.1003824 (PMC9597503; doi:10.3389/fmicb.2022.1003824)

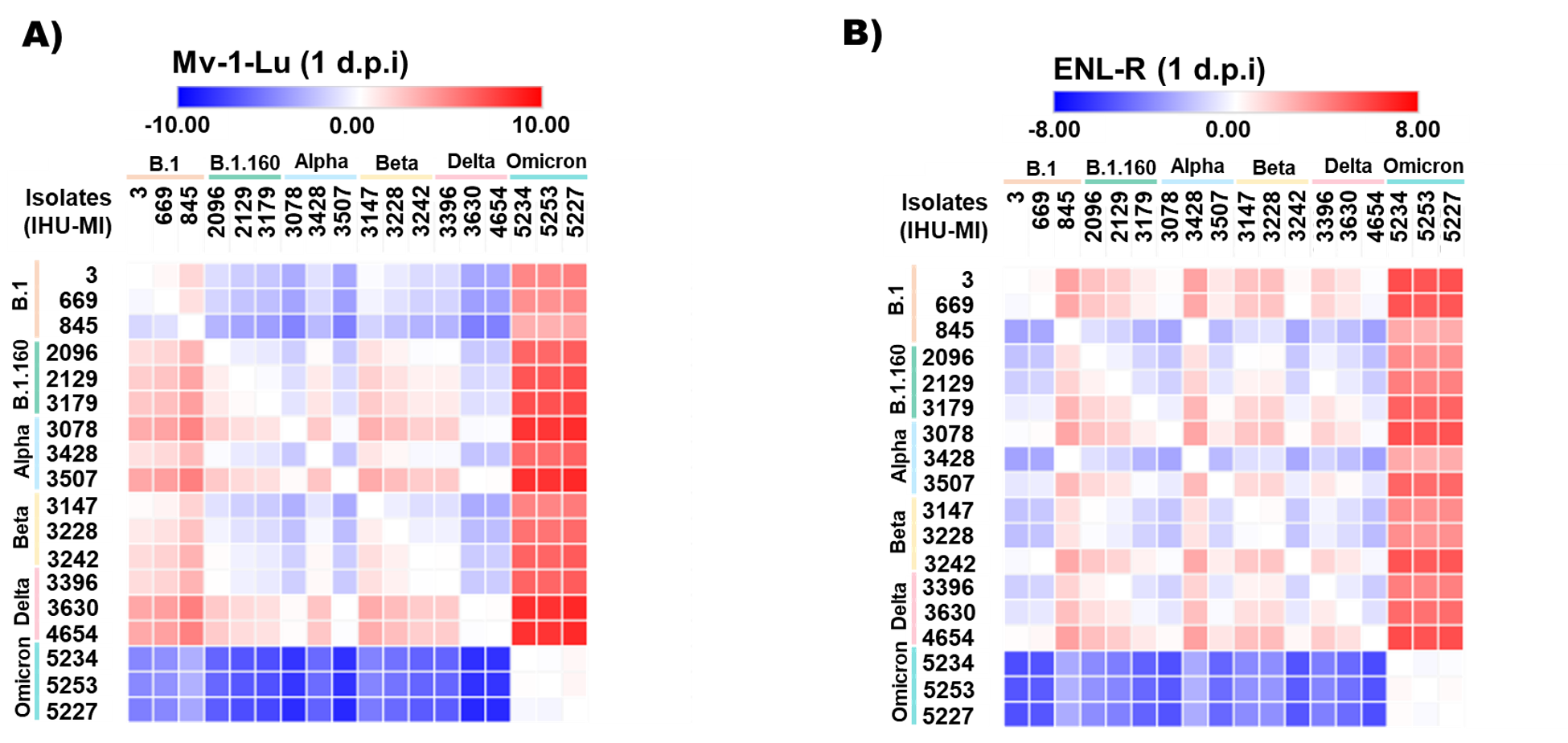

Supplement: SUPPLEMENTARY FIGURE 1 — Heatmap of replication rate among different SARS-CoV-2 isolates in mink lung cells 1-day post-infection. (A) Mv-1-Lu cells (B) ENL-R. [file Image_1.TIF]

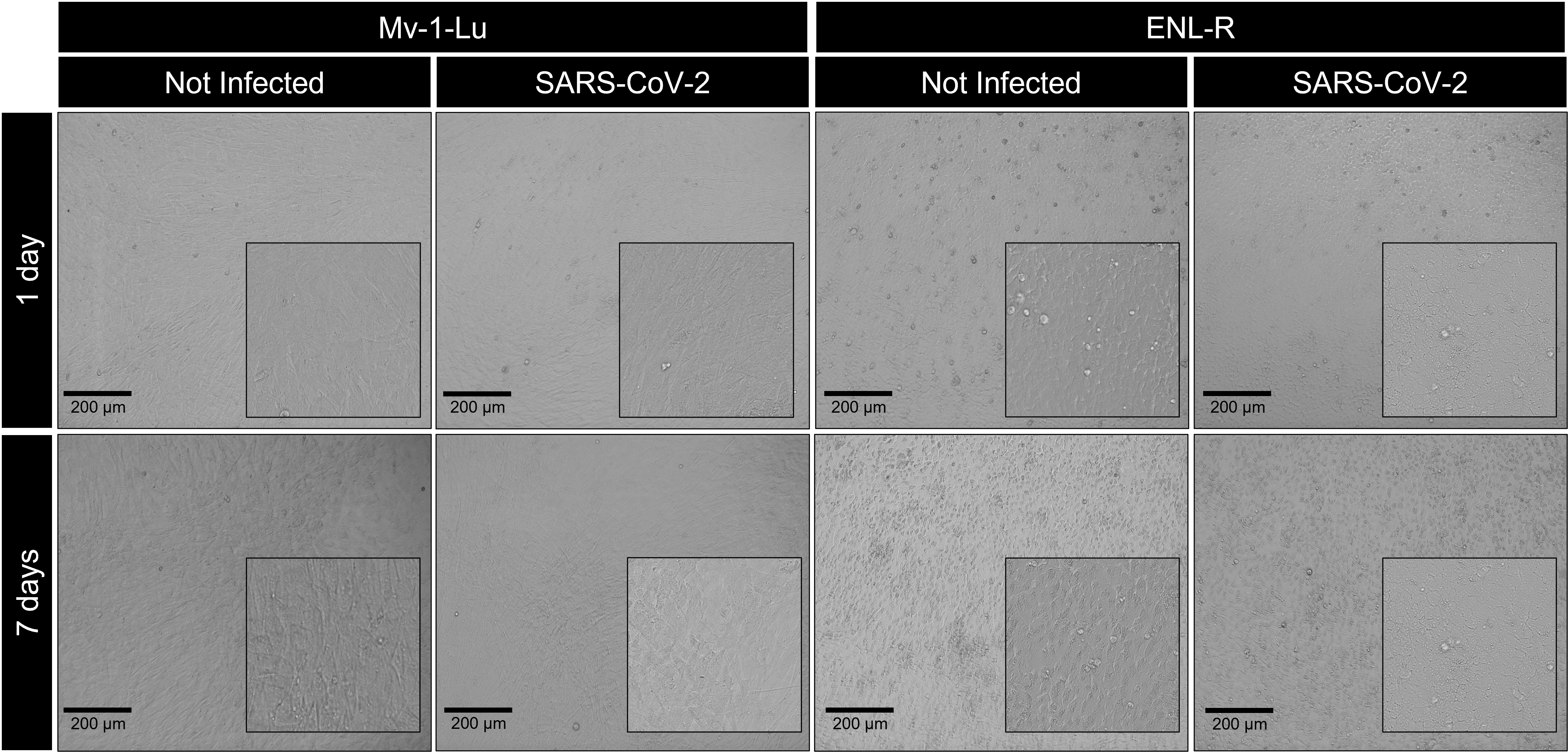

Supplement: SUPPLEMENTARY FIGURE 2 — Absence of cytopathic effect on mink lung cells after 7 days post-infection with SARS-CoV-2 (IHU-MI 3 Strain). [file Image_2.TIF]
